# Intestinal Microbiota is Different in Women with Preterm Birth: Results from Terminal Restriction Fragment Length Polymorphism Analysis

**DOI:** 10.1371/journal.pone.0111374

**Published:** 2014-11-05

**Authors:** Arihiro Shiozaki, Satoshi Yoneda, Noriko Yoneda, Rika Yonezawa, Takamichi Matsubayashi, Genichiro Seo, Shigeru Saito

**Affiliations:** 1 Department of Obstetrics and Gynecology, University of Toyama, Toyama, Japan; 2 Toa Pharmaceutical Co., Ltd., Tokyo, Japan; 3 Toa Pharmaceutical Co., Ltd., Gunma, Japan; Medical Faculty, Otto-von-Guericke University Magdeburg, Medical Faculty, Germany

## Abstract

Preterm birth is a leading cause of perinatal morbidity and mortality. Studies using a cultivation method or molecular identification have shown that bacterial vaginosis is one of the risk factors for preterm birth. However, an association between preterm birth and intestinal microbiota has not been reported using molecular techniques, although the vaginal microbiota changes during pregnancy. Our aim here was to clarify the difference in intestinal and vaginal microbiota between women with preterm birth and women without preterm labor. 16S ribosomal ribonucleic acid genes were amplified from fecal and vaginal DNA by polymerase chain reaction. Using terminal restriction fragment length polymorphism (T-RFLP), we compared the levels of operational taxonomic units of both intestinal and vaginal flora among three groups: pregnant women who delivered term babies without preterm labor (non-PTL group) (n = 20), those who had preterm labor but delivered term babies (PTL group) (n = 11), and those who had preterm birth (PTB group) (n = 10). Significantly low levels of *Clostridium* subcluster XVIII, *Clostridium* cluster IV, *Clostridium* subcluster XIVa, and *Bacteroides*, and a significantly high level of *Lactobacillales* were observed in the intestinal microbiota in the PTB group compared with those in the non-PTL group. The levels of *Clostridium* subcluster XVIII and *Clostridium* subcluster XIVa in the PTB group were significantly lower than those in the PTL group, and these levels in the PTL group were significantly lower than those in non-PTL group. However, there were no significant differences in vaginal microbiota among the three groups. Intestinal microbiota in the PTB group was found to differ from that in the non-PTL group using the T-RFLP method.

## Introduction

We harbor more than 100 trillion microbes in and on our body and these microbes constitute our microbiota [1], [2]. Although only a small part of this microbiota can be cultured in a medium, culture-independent analyses, like 16S ribosomal ribonucleic acid (rRNA)-based genomic analysis [3], [4] and metagenomic analysis [5], have recently revolutionized our understanding of the microbiota in our body. The microbiota in our intestine plays a major role in health and disease [1] in our body. The gut microbiota interacts with the immune system, providing signals to promote the maturation of immune cells and the normal development of immune functions [6], [7], [8].

Preterm birth (PTB) is the leading cause of perinatal morbidity and mortality in developing and developed countries [9]. The frequency of preterm births is about 12 to 13% in the USA and 5 to 9% in many other developed countries including Japan. Spontaneous PTB is regarded as a syndrome resulting from multiple causes, including intrauterine infection or inflammation, stress, socioeconomic environment, and uterine over-distension. Risk factors for PTB in Western countries include a previous preterm birth, black ethnicity, periodontal disease, low maternal body mass index, short cervical length, and an elevated cervical-vaginal fetal fibronectin concentration, while multiple pregnancy, short cervical length, part-time worker, steroid use for asthma or collagen disease, low educational level, and male fetus were shown to be risk factors for PTB in Japan [10]. Although antibiotic treatment can eradicate bacterial vaginosis (BV) in pregnancy, the overall risk of PTB in pregnant women with BV was found not to be significantly reduced by antibiotic treatment [11].

An association between preterm birth and intestinal microbiota has not been reported using molecular techniques, although the vaginal microbiota changes during pregnancy. Aagaard et al. [12] showed that the vaginal microbiome signature in pregnancy was distinct from that in non-pregnant women with variation of taxa across vaginal subsites and depending on gestational age. Romero et al. reported that, in a longitudinal study, *Lactobacillus* spp. were the predominant members of the microbial community in normal pregnancy [13], and they did not detect a difference in human vaginal microbiota between women who subsequently had a spontaneous preterm delivery and those who delivered at term [14]. Koren et al. described dramatic remodeling of the gut microbiota over the course of pregnancy [15]. These changes of intestinal microbiota induced insulin resistance during pregnancy. Although abnormal vaginal microbiota such as BV was studied using 16S rDNA-based genomic analysis [16], there are no reports on a link between PTB and gut microbiota as determined by genomic analysis.

Terminal restriction fragment length polymorphism (T-RFLP) analysis has used non-targeted approaches to identify differences and similarities in microbial communities, but it does not provide direct sequence information. Because of its relative simplicity, T-RFLP analysis has been applied to the analysis of bacterial 16S rRNA genes and provides a facile means to assess changes in microbial communities [17]. In this prospective and cross-sectional study using T-RFLP analysis, we examined bacterium-derived 16S rRNA genes in feces and vaginal discharge to determine whether the microbiota differs among three groups as follows: pregnant women who delivered term babies without preterm labor (non-PTL group), those who had preterm labor but delivered term babies (PTL group), and those who had preterm birth (PTB group).

## Materials and Methods

This study is a prospective and cross-sectional study. The study protocol and informed consent documents were reviewed and approved by the University of Toyama Institutional Review Board. Written informed consent was obtained from all subjects prior to participation in the study. From 2011 to 2013, the participants were categorized into three groups as follows: (1) pregnant women who delivered term babies without preterm labor during pregnancy (non-PTL group) (n = 20), (2) those who had regular uterine contraction and received tocolytic agents but delivered term babies (PTL group) (n = 11), and (3) those who had regular uterine contractions, received tocolytic agents, and finally delivered preterm babies (PTB group) (n = 10). Women who were at earlier than 22 weeks of gestation and those who took any antibiotics, tocolytics and steroids during pregnancy were excluded. Clinical characteristics of all participants are shown in Table 1. Vaginal samples were obtained from the posterior vaginal fornix using a swab. Swabs were immersed in vaginal discharge for 10 seconds, then immediately placed into 3 mL of a buffer medium (100 mM Tris-HCl, pH 9.0, 40 mM Tris-EDTA, pH 8.0, and 4 M guanidine thiocyanate), and stored in a freezer at −20°C. Fecal samples were collected at home or our hospital by the participants. Briefly, the toilet was covered with a sterile sheet of paper, which was temporarily waterproof but would dissolve in the water of the toilet within a few minutes and could be flushed away with the remaining stool. The defecated fecal samples were quickly collected using a sterile spoon or a swab, immediately placed in 3 mL of buffer medium, and stored in a freezer at −20°C.

**Table 1:** Clinical characteristics of all participants.

Characteristics	Non-PTL (N = 20)	PTL (N = 11)	PTB (N = 10)	Analysis of variance Bonferroni’s multiple comparison
Maternal age (year), median (range)	34.0 (27–41)	30.5 (22–37)	33.4 (22–41)	*P* = 0.2134
Gestational weeks at sample collection, average (range)	28.6 (23–34)	28.5 (22–33)	28.0 (22–34)	*P* = 0.9351
Gestational weeks at birth, average (range)	39.2 (37–41)	37.9 (37–40)	33.4 (27–36)	*P* = 0.0000
Previous PTB, percent (n/N)	10.0 (2/20)	9.1 (1/11)	10.0 (1/10)	*P* = 0.7897
Smoking during pregnancy, n/N	0/20	0/11	0/10	-
Parity number, average (range)	0.9 (0–2)	1.1 (0–2)	0.4 (0–2)	*P* = 0.1651
Nulliparous, percent (n/N)	40.0 (8/20)	27.3 (3/11)	70.0 (7/10)	*P* = 0.3169
BMI at sample collection, average (range)	23.3 (17.6–29.1)	23.2 (18.9–29.4)	21.7 (17.0–27.2)	*P* = 0.3263

BMI: Body mass index (kg/m^2^).

non-PTL: non-preterm labor.

PTL: preterm labor.

PTB: preterm birth.

### Terminal restriction fragment length polymorphism (T-RFLP) analysis

In order to investigate the microbiota of the fecal and vaginal samples obtained from all the subjects, terminal restriction fragment length polymorphism (T-RFLP) analysis was performed, as previously reported [18].

Fecal and vaginal samples were suspended in a solution containing 100 mM Tris-HCl, pH 9.0, 40 mM Tris-EDTA, pH 8.0, and 4 M guanidine thiocyanate, and kept at −20°C until deoxyribonucleic acid (DNA) extraction. An aliquot of 0.8 mL of the suspension was homogenized with zirconia beads in a 2.0 mL screw cap tube using a FastPrep FP120A Instrument (MP Biomedicals, Irvine, CA) and placed on ice. After centrifugation (at 5000×g, for 1 min), the supernatant was transferred to the automated nucleic acid isolation system 12GC (Precision System Science, Chiba, Japan). Thereafter, DNA was extracted from the bead-treated suspension using the Magtration-MagaZorb DNA Common Kit 200 N (Precision System Science, Chiba, Japan).

The 16S rDNA was amplified from human fecal DNA using fluorescent-labeled 516f primer (5′-(6-FAM)-TGCCAGCAGCCGCGGTA-3′) and 1492r primer (5′-GGTTACCTTGTTACGACTT-3′) and from human vaginal DNA using fluorescent-labeled 27f (5′-(6-FAM)-AGAGTTTGATCCTGGCTCAG-3′) and 1492r primer (5′-GGTTACCTTGTTACGACTT-3′), with Hot-starTaq DNA polymerase using Gene Amp PCR system 9600 (Applied Biosystems, CA, USA). The amplification program used was as follows: preheating at 95°C for 15 minutes; 30 cycles of denaturation at 95°C for 30 seconds, annealing at 50°C for 30 seconds, and extension at 72°C for 1 minute; and finally terminal extension at 72°C for 10 minutes. The amplified DNA was purified using a MultiScreen^R^ PCRµ96 Filter Plate (Millipore, MA, USA) and verified by electrophoresis.

The restriction enzymes were selected according to Nagashima *et al*. [19]. In brief, the PCR product purified from fecal samples was digested with 10 U of *Bsl* I (New England BioLabs, Inc., Ipswich, USA) at 37°C for 3 hours or that from vaginal samples with 10 U of *Msp* I (TAKARA, Shiga, Japan) at 37°C for 3 hours. The resultant DNA fragments, namely, fluorescent-labeled terminal restriction fragments (T-RFs), were analyzed using an ABI PRISM 3130xl genetic analyzer, and their length and peak area were determined using the genotype software GeneMapper (Applied Biosystems).

According to the methods described by Nagashima *et al.* [19], the T-RFs were divided into 29 operational taxonomic units (OTUs) for fecal samples or 22 OTUs for vaginal samples. The OTUs were quantified as the percentage values of individual OTU per total OTU areas, which were expressed as the percent of the area under the curve (%AUC). The bacteria predicted for each classification unit OTU were identified with reference to Human Fecal Microbiota T-RFLP profiling (http://www.tecsrg.co.jp/t-rflp/). To minimize inter- and intra-observer coefficient of variations of the OTU, 1 author (TM) conducted all T-RFLP analyses.

For objective interpretation of the difference in T-RF patterns, cluster analyses were performed using the software SPSS (IBM Statistics, ver. 20.0, NY, USA). T-RF patterns produced by digestion with restriction enzymes (*Bsl* I or *Msp* I) were quantified as the proportion of the total peak area of all T-RFs. The levels of similarity among fecal and vaginal samples were calculated as correlation coefficients, and represented graphically by a scatter plot using principal component analysis.

### Statistical analysis

All values of OTU are expressed as the mean ± standard error (SE). Comparisons of quantitative data among the three groups were carried out by a multiple comparison technique (Bonferroni). False discovery rates were calculated using Benjamini-Hochberg method. Differences were considered significant at *P* values of less than 0.05. For multivariate analysis of the data, principal component analysis and cluster analysis were used. The principal component analysis was performed unsupervised.

## Results

Of 21 women with preterm labor (the PTL group plus the PTB group), 10 women (47.6%) finally delivered their babies before 37 weeks of gestation.

### Principal component analysis (PCA)

#### Fecal samples

**Figure 1:**
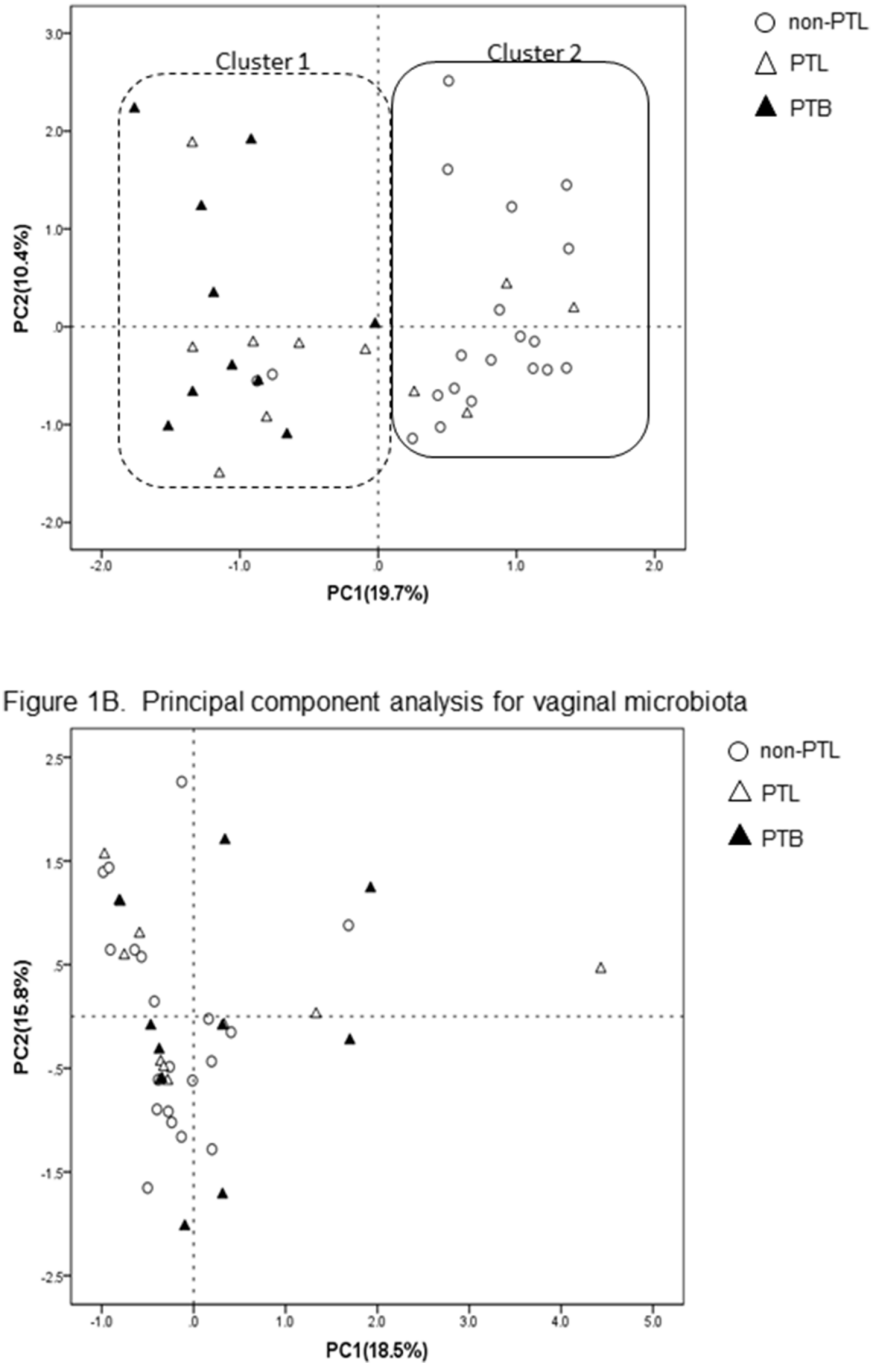
A. Principal component analysis of fecal microbiota. Principal component analysis scores are plotted based on the relative abundance of OTUs of vaginal microbiota. The percentage of variation explained by the principal coordinates is indicated on the axis. Open circles (○) represent the non-PTL group, open triangles (△) the PTL group, and closed triangles (▴) the PTB group. A dotted line, on the left in Figure 1A, shows ‘cluster 1′, which contains all 10 cases of the PTB group, as well as 2/20 of the non-PTL group and 7/11 of the PTL group. A solid circle, on the right in Figure 1A, shows 90% of the non-PTL group (18/20) and 36.4% of the PTL group (4/11). The PTL group occupied an intermediate position between the non-PTL group and the PTB group. B. Principal component analysis of vaginal microbiota. Principal component analysis scores are plotted based on the relative abundance of OTUs of vaginal microbiota. The percentage of variation explained by the principal coordinates is indicated on the axis. Open circles (○) represent the non-PTL group, open triangles (△) the PTL group, and closed triangles (▴) the PTB group.

PCA was performed based on the relative abundance of OTUs in fecal samples. The first two principal component scores, which accounted for 19.7% and 10.4% of the total variation, were calculated. Hierarchical clustering of fecal samples on the basis of their first two principal component scores separated the fecal samples into two primary clusters (Figure 1A). One cluster (labeled as ‘cluster 1′, thick dotted line, left in Figure 1A) included all 10 cases of the PTB group, as well as 2/20 of the non-PTL group and 7/11 of the PTL group. The other cluster (labeled as ‘cluster 2′, thick solid line, right in Figure 1A) was dominated by 90% of the non-PTL group (18/20) and 36.4% of the PTL group (4/11). No PTB cases were included in cluster 2 (0/10). The components of the PTL group occupied an intermediate position between the non-PTL group and the PTB group.

#### Vaginal samples

In vaginal samples, the first two principal component scores were calculated (the first and second principal components accounted for 18.5% and 15.8% of the total variation, respectively) (Figure 1B). The PCA showed that there was similarity among these three groups.

### Terminal restriction fragment length polymorphism profile

The percentages of OTU (percentage values of individual OTU area per total OTU) of representative OTUs for fecal samples after *Bsl* I digestion are shown in Table 2 and those for vaginal samples after *Msp* I digestion in Table 3.

**Table 2:** Average OTU sores in fecal samples.

OTU	Bacteria predicted by T-RF length	Non-PTL (N = 20)	PTL (N = 11)	PTB (N = 10)	Bonferroni’sadjustment*P* value	False DiscoveryRate *P* value
106	*Clostridium* subcluster XIVa	1.85±0.75	0.33±0.27	0.19±0.13	NS	NS
110	*Clostridium* cluster IV	1.25±0.42	3.89±2.99	3.03±1.30	NS	NS
124	*Bifidobacterium*	11.19±1.98	14.10±4.08	5.91±2.61	NS	NS
168	*Clostridium* cluster IV	0.52±0.15	0.18±0.18	0.08±0.08	NS	NS
317	*Prevotella*	4.58±2.79	0.31±0.16	0.99±0.60	NS	NS
332	*Lactobacillares*	1.29±0.34	1.91±0.60	0.87±0.42	NS	NS
338	*Clostridium* cluster IV	0.57±0.16	0.37±0.19	0.00±0.00	NS	NS
366	*Bacteroides*	3.56±1.13	4.62±1.96	5.71±2.38	NS	NS
369	*Clostridium* cluster IV	0.27±0.18	0.89±0.86	2.76±1.32	NS	NS
469	*Bacteroides*	37.10±3.33	30.44±5.66	22.76±4.44	NS	NS
494	*Clostridium* subcluster XIVa	5.90±1.51	10.49±4.46	10.51±5.04	NS	NS
520	*Lactobacillares*	0.79±0.33	0.18±0.12	0.00±0.00	NS	NS
650	*Clostridium* cluster XVIII	2.12±0.34	0.70±0.35	0.26±0.21	Non-PTL vs. PTB, *P* = 0.0016; non-PTLvs. PTL, *P* = 0.0150	Non-PTL vs. PTB, *P* = 0.0125; non-PTL vs. PTL, *P* = 0.0449
657	*Lactobacillares*	5.18±2.54	17.63±8.29	24.24±7.17	Non-PTL vs. PTB, *P* = 0.0497	NS (*P* = 0.1089)
749	*Clostridium* cluster IV	6.41±1.15	2.90±1.22	0.80±0.80	Non-PTL vs. PTB, *P* = 0.0060	Non-PTL vs. PTB, *P* = 0.0289
754	*Clostridium* subcluster XIVa	1.70±0.28	1.63±1.03	0.00±0.00	NS	NS
853	*Bacteroides*	0.84±0.13	0.60±0.27	0.07±0.07	Non-PTL vs. PTB, *P* = 0.0087	Non-PTL vs. PTB, *P* = 0.0348
919	*Clostridium* cluster XI, *Clostridium* subcluster XIVa	1.30±0.23	0.36±0.16	2.06±0.55	PTL vs. PTB,*P* = 0.0047	PTL vs. PTB, *P* = 0.0285
940	*Clostridium* subcluster XIVa, *Enterobacteriales*	3.79±0.68	3.62±1.10	11.43±3.72	NS	NS
955	*Clostridium* subcluster XIVa	5.37±0.64	1.72±0.80	0.34±0.34	non-PTL vs. PTB, *P* = 0.0000; non-PTLvs. PTL, *P* = 0.0012	non-PTL vs. PTB, *P* = 0.0005; non-PTL vs. PTL, *P* = 0.0125
990	*Clostridium* subcluster XIVa	3.61±0.69	2.87±0.86	7.98±6.66	NS	NS
Others		0.83±0.42	0.26±0.20	0.00±0.00	NS	NS

OTU: operational taxonomic unit,

T-RF: terminal restriction fragment.

non-PTL: non-preterm labor.

PTL: preterm labor.

PTB: preterm birth.

**Table 3:** Average OTU scores in vaginal samples.

OTU	Bacteria predicted by T-RF length	non-PTL (N = 20)	PTL (N = 11)	PTB (N = 10)	*P* value
57	Unknown	8.78±1.77	12.58±4.12	10.12±2.61	NS
74	*Enterococcus*	0.47±0.27	0.36±0.31	0.72±0.49	NS
93	*Chitinophagaceae*	1.20±0.53	4.25±2.74	4.16±2.41	NS
98	*Prevotella*	0.54±0.24	2.39±2.19	5.45±2.30	NS
134	*Bifidobacterium*	0.89±0.86	0.05±0.05	2.23±1.78	NS
147	*Firmicutes*	2.37±1.56	0.28±0.13	3.94±3.55	NS
163	*Actinobacteria*	3.17±1.67	0.01±0.01	5.78±4.93	NS
170	*Firmicutes*	0.33±0.17	0.74±0.35	0.53±0.28	NS
179	*Lactobacillus*	27.42±8.09	34.34±12.45	25.40±8.72	NS
189	*Lactobacillus*	43.41±8.38	39.68±13.11	29.01±10.90	NS
230	*Firmicutes*	0.41±0.15	0.59±0.38	0.37±0.25	NS
281	*Actinobacteria*	6.34±4.12	1.16±1.04	2.95±2.95	NS
300	*Clostridiales*	1.03±0.59	0.08±0.08	5.73±4.40	NS
563	*Streptococcus*	0.18±0.15	0.78±0.78	0.92±0.41	NS
573	*Lactobacillus*	3.24±1.29	2.39±1.35	2.62±0.91	NS
Others		0.20±0.15	0.33±0.33	0.05±0.05	NS

OTU: operational taxonomic unit.

T-RF: terminal restriction fragment.

non-PTL: non-preterm labor.

PTL: preterm labor.

PTB: preterm birth.

#### Fecal samples

When comparing respective OTUs of fecal samples between the non-PTL group and the PTB group, the peak areas of the OTU 650, OTU 657, OTU 749, OTU 853, and OTU 955 (digested with *Bsl* I) were significantly different (Table 2). The amounts of *Clostridium* cluster XVIII (OTU 650), *Clostridium* cluster IV (OTU 749), *Bacteroides* (OTU 853), and *Clostridium* subcluster XIVa (OTU 955) were significantly lower in the PTB group than those in the non-PTL group (*P* = 0.0125, *P* = 0.0289, *P* = 0.0348, and *P* = 0.0005, respectively). The amounts of *Clostridium* cluster XVIII and *Clostridium* subcluster XIVa in the PTL group were significantly higher than those in the non-PTL group (P = 0.0449 and P = 0.0125, respectively).

When comparing respective OTUs between the PTL group and the PTB group, the amount of *Clostridium* cluster XI and *Clostridium* subcluster XIVa (OTU 919) was significantly lower in the PTB group than that in the PTL group (*P* = 0.0285).

#### Vaginal samples

In contrast, the % OTU for vaginal samples after *Msp* I digestion from the non-PTL group was quite similar to that from the PTL or the PTB group (Table 3).

## Discussion

In this study, we first showed that the fecal microbiota in the PTL group was substantially different from that in the non-PTL group using T-RFLP analysis. Average scores of OTU 650, OTU 749, OTU 853, and OTU 955 were significantly lower in the fecal microbiota from the PTB group than those from the non-PTL group. Nagashima *et al*. [19] reported that OTU 749 and OTU 955 most likely represent *Clostridium* cluster IV species and *Clostridium* subcluster XIVa species, respectively, as determined by the 16S rDNA clone library method. Taking these findings together, it was revealed that the levels of *Clostridium* cluster XVIII, *Clostridium* cluster IV, *Clostridium* subcluster XIVa, and *Bacteroides* were significantly reduced in the fecal microbiota from the PTB group. Our result first showed that there was a significant difference in the average OTU scores of *Clostridium* cluster XVIII, *Clostridium* cluster IV, and *Clostridium* subcluster XIVa between mothers with non-PTL and those with PTB, while mothers with PTL had intermediate scores between them. This finding does not mean that the intestinal microbiota accidentally changed in the case of the PTB group, but rather it suggests that some alterations in intestinal microbiota are associated with the clinical findings of mothers with PTB.

Our result is compatible with the report by Romero, who found no differences in human vaginal microbiota between women who subsequently had a spontaneous preterm delivery and those who delivered at term. In addition, more recent studies have shown that bacteria from the oral cavity are most often found in the amniotic fluid of patients with preterm labor, demonstrating that periodontal pathogens/byproducts may reach the placenta and spread to the fetal circulation and amniotic fluid [20]. Taken together, these findings may suggest that oral and/or intestinal, not vaginal, microbiota could induce pathogens and the secretion of elevated levels of inflammatory mediators, which in turn may cause premature birth or suggest that dysbiosis caused by oral and/or intestinal, not vaginal, microbiota may render the uterus and/or the placenta susceptible to infection.

Recently, bacterial vaginosis-associated bacteria (BVAB) in the order *Clostridiales:* BVAB1, BVAB2, and BVAB3, were identified in association with bacterial vaginosis [21]. Subsequently, Foxman et al. reported that, in vaginal fluids, one of the Clostridia-like bacteria, BVAB-3, was consistently associated with a reduction in the risk of preterm birth for all ethnic groups (risk ratio, 0.55; 95% confidence interval, 0.39–0.78) [22]. Their result is compatible with our findings in that the level of *Clostridium* was significantly lower in preterm birth, although its level in our studies was lower in the samples not from vaginal fluid but from feces.

Among the microbiota indigenous to the murine and human colon, the genus *Clostridium* belonging to clusters XIVa and IV is reported to be an outstanding inducer of colonic CD4^+^ CD25^+^ Foxp3^+^ regulatory T (Treg) cells [7], [8]. *Bacteroides fragilis* has also been shown to induce Treg cells in mouse [23]. It was suggested that polysaccharide A (PSA) produced by *Bacteroides fragilis* potentiates the suppressor activity of Treg cells. The balance between beneficial and potentially harmful species in the commensal microbial community, known as dysbiosis, has often been linked to the development of inflammatory bowel disease (IBD) in humans and analogous intestinal inflammation in mice [18]. Importantly, intestinal Treg cells play a key role in regulating inflammation by the production of IL-10 [8], [23], [24], [25] and decreased intestinal Treg cells were observed in IBD patients. Preterm labor may be viewed as inflammation caused by inappropriately expanded components of the normal bacterial community. In IL-10 knockout mice, a very small amount of lipopolysaccharide (LPS) induces preterm delivery [26]. These findings suggest that decreased intestinal *Clostridium* cannot induce an adequate volume of Treg cells, resulting in susceptibility to inflammation. Indeed, decreased Treg cell volume and function in the peripheral blood have been reported in preterm labor [27], [28], [29], [30], [31], although the level of intestinal Treg cells is unclear in these papers and this study. Further studies are needed to clarify the relationship between intestinal microbiota and peripheral Treg cells or intestinal Treg cells.

The peak area of the 919-bp OTU (*Clostridium* cluster XI, *Clostridium* subcluster XIVa) in the PTB group was significantly larger than that in the PTL group (*P* = 0.0285). This is a limitation of the T-RFLP method. We should thus reevaluate the bacterial species using metagenomic analysis.

Several limitations were identified in this study. Because of the small number of cases (fewer than 30) in each group, information that can only come from patient participation in well-designed clinical trials is needed to improve the management of PTB. Second, we did not check the level of Treg cells in peripheral blood and therefore could not compare the relationship between the fecal microbiota of the mother and the level of Treg cells. Further investigation is needed to address whether the difference in gut microbiota is related to the expression of immunoregulatory Treg cells or inflammation-induced Th17 cells that are induced by gut *Clostridium* strains and segmented filamentous bacteria, respectively. Third, it is possible that the unidentified confounders may have an effect on changes in the microbial communities. An alternative interpretation is that, as Aagaard et al. [12] reported, the vaginal microbial 16S rRNA gene catalogue may uniquely differ in pregnancy, with variation of taxa across vaginal subsites and depending on gestational age. Further study is required to determine the relationship between the difference in average OTU for some phylotypes and PTB. Fourth, owing to the cross-sectional nature of the study, our finding about the causal relationships between fecal microbiota and PTB should be interpreted with caution. Fifth, we didn’t measure the concentrations of organic acids, indole, and ammonia. And we also didn’t measure pH and moisture and didn’t compare these values with those from normal pregnancy. Sixth, the selection of optimal T-RFLP probes is a subject of considerable ongoing discussion in the field of microbiome research. T-RFLP analysis only identify differences and similarities in microbial community and the resulting OTUs aren’t assigned to a specific species. Unfortunately, there is no perfect set of T-RFLP probes for fecal and vaginal microbiome. Yet, the T-RFLP data can readily be analyzed using various statistical algorithms to quantitatively ascertain similarities and differences among communities and to infer plausible community of intestine and vagina. Seventh, we are unable to grasp the importance of the results that patients had preterm babies had a higher average OTU for Lactobacillares phylotypes than those who delivered term babies. Yet, the identification of significant differences in average OTUs provides evidence that the study of the intestinal microbiota during pregnancy can yield important insights into the relationship between the fluctuation of microbial communities and adverse pregnancy outcome like PTB. Further studies are required to confirm this finding and elucidate the role of intestinal microbiota in PTB. Eighth, the principal component analysis is not a prefect technique to analyze the T-RFLP data. Therefore, the performance of principal component analysis should be interpreted with caution since there were no validation samples to test.

Many studies have been performed to identify the differences in microbial diversity between healthy individuals and patients with rheumatoid arthritis (RA) [32], inflammatory bowel disease (IBD) [33], and type 1 diabetes [34]. However, the results about the fecal level of *Clostridium* in these patients are conflicting (decrease [32], no change [33], and increase [34]). When abnormality in fecal microbiota in the PTL group is found, the question arises of whether microbiotic alteration, dysbiosis, is a cause or a result of preterm delivery. We speculate that the changes in the immune system through fecal dysbiosis may change the uterine activity. In addition, the underlying mechanisms resulting in alteration of the microbiota remain to be clarified. Recently, intake of probiotic food was found to be associated with a reduced risk of spontaneous preterm delivery and preeclampsia [35], [36]. Prospective studies are needed to clarify whether intestinal dysbiosis before pregnancy might cause uterine inflammation and induce uterine contraction or cervical ripening during pregnancy.

## Conclusion

Disturbance of the intestinal flora, dysbiosis, during pregnancy was first observed in the PTB group in this study. This may cause inflammatory reactions in the uterus leading to PTB. Further study is needed to clarify the relationship between PTB and dysbiosis of intestinal bacterial flora.
